# Terrestrial medium and large-sized mammalian species diversity in Michole Community Protected Forest, southern Ethiopia

**DOI:** 10.1186/s40850-022-00121-0

**Published:** 2022-04-24

**Authors:** Amanuel Agebo, Wondimagegnehu Tekalign

**Affiliations:** Department of Biology, Wolaita Sodo University, College of Natural and Computational Sciences, PO Box 138, Wolaita Sodo, Ethiopia

**Keywords:** Abundance, Anthropogenic challenges, Conservation measures, Diversity, Community-based forest, Wildlife species

## Abstract

The study on medium and large-sized mammalian species' diversity and distribution is important for conservation efforts in the different protected areas of Ethiopia. The present study was intended to investigate the species diversity of medium and large-sized mammalian fauna between October 2019 and July 2020 in Michole Community Protected Forest, southern Ethiopia. The study was conducted by stratification of the study area into three habitat types: riverine forest (3.37 km^2^), woodland (4.14 km^2^), and grassland with scattered trees (2.33 km^2^) based on the vegetation cover. A diurnal transect survey method was implemented to record the mammalian species diversity. A total of 18 representative sample transect lines (six in riverine forest, four in grassland with scattered trees, and eight in woodland habitats) that varied in length and width were used. A total of 17 species of medium and large-sized mammalian species were identified and recorded in the study area. As a result, the orders Carnivora and Primates have the greatest abundance, while the order Lagomorpha has the least. Anubis baboon (*Papio anubis*) was the most abundant species (15.14%), followed by Spotted hyena (*Crocuta crocuta*) (12.98%), Crested porcupine (*Hystrix cristata*) (12.51%), Vervet monkey (*Chlorocebus aethiops*) (10.35%), Common duiker (*Sylvicapra grimmia*) (8.80%), and Giant root-rat (*Tachyoryctes macrocephalus*) (8.65%). The distributions of mammals among the three habitat types were comparable. The riverine forest harbored the highest mammalian diversity index (H′ = 2.35) followed by the woodland (H′ = 2.32), and the grassland with scattered trees (H′ = 2.30), respectively. The greatest species similarity was recorded in woodland (0.902). The study area harbors considerable mammalian species that are threatened by interacting anthropogenic factors. So, urgent conservation measures by concerned sectors are needed to safeguard these animals and their habitat.

## Introduction

Mammalian species are thought to be indicator and umbrella species of the terrestrial ecosystems because they help to conserve other species and maintain ecosystem balance [1–3]. Particularly, the medium and large-sized mammals are key components of forest and savannah communities and are therefore considered good indicators of ecosystem health [4]. Anthropogenic activities, such as increased hunting rates, habitat loss, habitat degradation, habitat fragmentation, increased human settlement, and urbanization, are the main threats to mammalian species [5–8]. Nowadays, numerous anthropogenic factors have promoted habitat loss and fragmentation, and the decline and losses of global mammalian biodiversity [9]. Conflict occurs when the requirements of wildlife animals overlap with those of human populations [10]. The challenges are particularly severe in Sub-Saharan African countries, which are at present undergoing rapid population growth [11]. Due to subsistence livelihood, many of the people of Africa are exerting pressure on the wildlife, especially the mammalian species [12]. Knowledge of the causes and consequences of human-wildlife conflict at the human-wildlife interface is essential for developing effective conservation plans that benefit both people and wildlife [13].

In particular, in the Horn of Africa, Ethiopia, the profound topography and climate are the most significant predictors of the high mammalian diversity [14–16], and the highest mammalian species level of endemicity [17], in which heterogeneous habitats support different species of mammals [18]. Currently, 320 mammalian species have been recorded, and 55 of them are considered to be endemic to the country [19]. At the moment, many of the protected areas and/or forests of Ethiopia are facing many challenges, mainly due to the growing human population, border conflicts, and recurring drought.

Many of the studies on terrestrial mammalian species in Ethiopia have been limited to the protected areas [20, 21]. However, there is some research evidence on the diversity and distribution of mammalian species outside the protected areas, such as in the communal areas and human-dominated landscapes [3, 8, 19, 22–27].

Although many forests exist in the southern parts of Ethiopia, the wildlife species diversity and other ecological aspects have not been well studied and documented. Thus, the current study was carried out in Michole Forest, which is one of the few forests in the Wolaita zone, southern Ethiopia; where there was no study carried out on the wildlife species. Moreover, there has been no ecological study on the wildlife diversity undertaken in the area until now. For the crucial conservation measures of the mammalian species in the area, knowledge about those species is significant. Therefore, the present study was aimed at identifying the diversity, estimating the abundance, and examining the habitat association and seasonal variation of the medium and large-sized mammalian species in the Michole Community Protected Forest, southern Ethiopia. During the study, the size of the mammalian species was categorized into medium-sized (2–15 kg), and large-sized (> 15 kg) mammals based on their body weight [28].

## Materials and methods

### The study area

Offa district is one of the 12 districts in the Wolaita Zone, southern Ethiopia. It is located 406 km south of Addis Ababa and 183 km away from Hawssa town, and 29 km away from Wolaita Sodo town, in the southern direction along the Goffa-Sawla road. Offa is bordered in the northwest by Kindo Koysha, in the northeast by Sodo Zuriya, in the south by Gamo and Gofa zones, in the west by Kindo Didaye, and in the east by the Humbo districts. The administrative town of Offa district is Gesuba. Currently, the area of the district covers 374.74 km^2^. Among them, 18,313 hectares of land are under cultivation, 7216 hectares are used for grazing, and 2278 hectares are covered by forests and bushes. Currently, the district is divided into 22 rural and two urban kebeles (the smallest administrative units)*.*

**Fig. 1:**
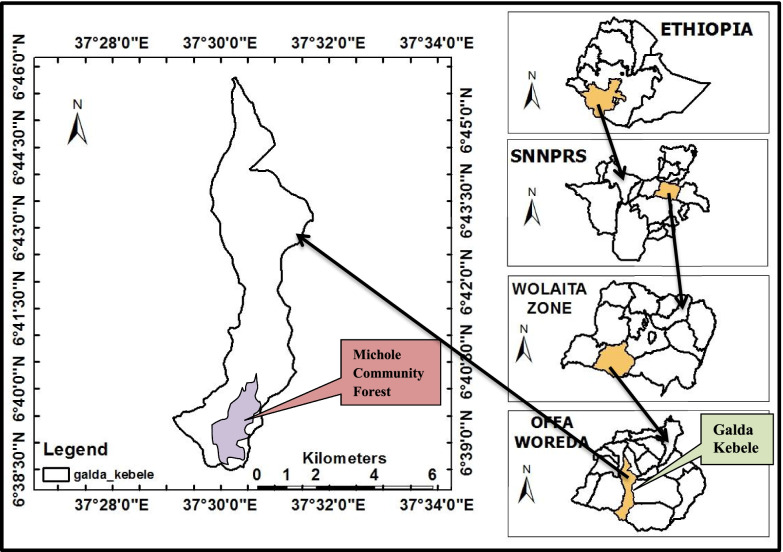
Map of the study area

The study area, Michole Community Forest, is located between 6^0^39′0″ − 6^0^45′0″N latitude and 37^0^27′30″ − 37^0^ 33′30″ E longitudes with altitudinal ranges from 1200 to 2028 m a.s.l. It is found in Wolaita Zone at a distance of 432 km from Addis Ababa, the capital city of the country, and 47 km southwest of Wolaita Sodo town, the zone administration seat (Fig. 1). The total area of the forest is 983.7 ha. The rainfall pattern is bimodal in type.

The district consists of three agro-climatic zones, and the elevation ranges between 1100 and 2800 m asl. The mean annual rainfall ranges between 800 mm and 1400 mm. The mean annual temperature ranges between 14 °C and 34 °C. Approximately, 2278 ha of the total land area are covered by forest. There are five major rivers and four streams that flow west to east in a direction, and all of them are Omo tributaries. Insufficient and irregular rain cause uncertainty in agricultural activity. The rainy season runs from June to August, and the remaining months are dry. The range of habitats in the Michole Community Forest is diverse in altitude and vegetation cover. This is woodland (414.1 hectares), which is the largest part of the forest area and is dominated by Acacia (*Acacia abyssinica*) and Cordia (*Cordia africana*) trees, grassland with scattered trees (232.7 hectares), which is dominated by the grass *Chrysopogon* (*Chrysopogan auyrcheri*) and is one of the main sources of food for grazing animals. The riverine forest (336.9 hectares) is covered by the large trees that grow all year and is dominated by Podocarpus (*Podocarpus flacatus*) and *Juniper* (*Juniperus procera*). Once the wild animal species existed in the area, some of them, including the known big mammals in the world like elephants and buffalos, have gone out of the district due to the traditional killing and hunting of wild animals. Such types of cultural practices were common in the Offa district.

According to the 2007 Central Statistics Agency (CSA) Report, the total population of the district is 120,548. Out of these numbers, 66,747 are males and 53,801 are females. Of the total 9299 urban dwellers, 4971 are males and 4328 are females. Based on this data; males’ outnumbered females in urban and rural areas. The combined number of males exceeded the population of the district. The population density of the district is 359 km^2^.

A preliminary survey was carried out in October 2019 in the study area. During this period, essential information such as accessibility, climatic conditions, vegetation type, fauna, topography, infrastructure, and anthropogenic activities in the area was gathered. Detailed studies were carried out from November 2019 to August 2020. The diversity, distribution and relative abundance of medium- and large mammalian species were quantified during both the dry (December–February) and wet (May–July) seasons.

The study area was stratified into three main study blocks using aerial photography (scale 1:30,000), satellite imagery, and area topography maps (scales 1:50,000 and 1:250,000) [5] (Kingdon, 2003). The boundaries of each study unit were traced and followed based on the main vegetation types of the study area. These include grassland, woodland, and riverine forest. For sampling, a variation of Norton-Griffiths' unequally sized sample unit ratio method was used. 

Out of the total blocks in the study area, several representative sample blocks were randomly selected. The sampling blocks selected from each habitat type represent 20–25% of each of the surveyed areas. Randomly selected transects were then established in each block. Data on the diversity, distribution, and abundance of medium and large-sized mammals in Michole Community Forest was collected through a randomly selected line transect survey. A total of 18 representative samples of transect lines (six in riverine forest, four in grassland, and eight in woodland habitats) that varied in length and width were used.

In the woodland, a transect length of 1.5 km and a width of 100 m were used, and in riverine forest, a transect length of 3 km and a width of 50 m was used, and in grassland with scattered trees, a transect length of 2 km and a width of 200 m was used, and the distance between each transect was 1 to 2 km to avoid double counting. Variation in the length and width of the transect line was determined by the type of vegetation cover and topography of the area (Table 1).

**Table 1:** The length and width of transects choosen at random

Habitats	Number of potential transects	Number of sample transects	Length and width of the transect (km)
Riverine forest	26	6	3 km x 0.5 km
Grassland	14	4	2 km x 0.2 km
Woodland	32	8	1.5 km x 0.1 km
Total	72	18	

## Methods

Direct and indirect observations of medium and large mammals were conducted along randomly selected transect lines (trails, footpaths, and other access routes). The data was collected with the help of 18 well-experienced local people during the wet and dry seasons at a constant speed to maximize the probability of seeing all individuals on the transect [29]. Transect counts were carried out twice a day for three days in each month (survey period) during both the dry and wet seasons, from 06:00 to 10:00 in the morning and 16:00 to 18:00 in the late afternoon, when the animals were active and visibility was good. The study area was surveyed about 36 times during both the dry and wet seasons. During counting, careful descriptions of the observed group/individuals were made based on natural markings of individuals for future identification and to avoid double-counting. The individuals observed per transect were pooled together and extrapolated to estimate the population for the whole study area.

The numbers of individuals of each species observed at each time and habitat type were recorded during the survey. Observations were made with the naked eye or aided by binoculars (7 x 50 mm) while evidence of tracks, scats, dung, dens, burrows, carcasses, feeding remains, scratches, feeds, beds, and calls were considered as indirect observations [30, 31].

Indirect pieces of evidence are very useful when surveying animals that are naturally rare, elusive, and found at low densities. Mammalian species identification was made by using standardized field guides [32].

### Data analysis

The diversity measures consider both the number of species and the distribution of individuals within those species across the entire community [18]. Thus, such measures as the number of species, the relative abundance of individuals, and diversity (a combination of richness and evenness) were taken into account during data analyses.

 SPSS version 20 software and Microsoft Excel-2010 were used to analyze the data. Appropriate statistical methods such as the Chi-square test and descriptive analysis were used to compute differences in the abundance of mammal species among habitats and the seasonal variations in species compositions in the study area. The species diversity and evenness of mammals in each area and season were determined by using the Shannon–Wiener entropy index (H′) and Simpson’s diversity index. Shannon-Wiener diversity index (H), Simpson diversity index (1-D), and evenness (J): The Shannon–Wiener diversity index assumes that all species are represented in a sample species and is calculated by its formula:

$$H\:=\:-\;\left(-\sum Pi\;\ln Pi\right)$$

Where,

H = denotes the Shannon-Wiener diversity index.

Pi = The fraction of individuals in i^th^ species.

ln = Natural logarithm.

Simpson’s diversity index was issued to assess the diversity of any population in which each number belongs to a unique species and calculated by the formula:

$$D=1-\frac{(\sum\mathrm n\;(\mathrm n-1))}{\mathrm N(\mathrm N-1)}$$

Where D = Simpson’s diversity index, *n* = number of individual species, N = total number of organisms, Ʃ = summation, Evenness is a measure of the relative abundance of different species that make up the richness of an area by the formula: E = H/H_max_, H_max_, = lnS, S = Number of species. The relative abundance of each species (observed medium and large mammals) in the three habitat types was computed using the formula: abundance = total number of individuals of a species/total number of individual species in the sampled habitat×100.

Observed mammalian species were categorized as common if they were realized during the whole study period, uncommon if they were seen in more than half of the surveys, and rare if they were seen in less than half of the surveys. A simpson similarity index (SI) was also computed to assess the similarity between the three habitats regarding the composition of species.

$$SI\;=\;3C/\;I\;+\;II\;+\;III$$

Where: SI = Simpson’s similarity index; C = the number of common species in all tree habitats; I = the number of species in habitat one (riverine forest); II = the number of species in habitat two (woodland); and III = the number of species in habitat three (grassland).

## Results

### Medium and large-sized mammal diversity

During this study, 647 observational records of medium and large-sized mammals belonging to 17 species and grouped into 11 families and five orders, such as Primates, Artiodactyla, Rodentia, Carnivora, and Lagomorpha, were documented. Among the species are the Anubis baboon (*Papio anubis*), the Vervet monkey (*Cercopithecus aethiopus*), the Colobus monkey (*Colobus guereza)*, the Common duiker (*Sylvicapra girmma)*, the Bushbuck (*Tragelaphus sylvaticus)*, the Bushpig (*Potamochoerus larvatus)*, the Giant root-rat (*Tachyoryctes macrocephalus)*, the Crested Porcupine (*Hystrix cristatea)*, the Common jackal (*Canis aureus)*, the Spotted hyena (*Crocuta crocuta)*, the African civet (*Civettictis civetta*), the Common genet (*Genetta abyssinica*), the White-tailed mongoose (*Ichneumia albicauda*), the African wild cat (*Felis lybica*), the Serval cat (*Felis serval*), the Lion (*Panthera leo*), and the Abyssinian hare (*Lepus habessinicus*) (Table 2).

**Table 2:** The diversity of medium and large-sized mammalian species found in the Michole Community Forest

Order	Family	Species	Common name	IUCN category (Conservation status)	Season		Total
					Dry	Wet	
Primates	Cercopithecidae	*Papio anubis*	Anubis baboon	LR/lc	45	53	98
		*Cercopithecus aethiopus*	Vervet monkey	LR/lc	31	36	67
		*Colobus guereza*	Colobus monkey	LR/lc	8	13	21
Artiodactyla	Bovidae	*Sylvicapra girmma*	Common duiker	LR/lc	26	31	57
		*Tragelaphus sylvaticus*	Bushbuck	LR/lc	5	11	16
	Suidae	*Potamochoerus larvatus*	Bushpig	LC	11	17	28
Rodentia	Spaclacidae	*Tachyoryctes macrocephalus*	Giant root-rat	EN	30	26	56
	Hystricidae	*Hystrix cristatea*	Crested Porcupine	LC	37	44	81
Carnivora	Canidae	*Canis aureus*	Common jackal	LC	17	22	39
	Hyaenidae	*Crocuta crocuta*	Spotted hyena	LC	51	33	84
	Viverridae	*Civettictis civetta*	African civet	LC	7	10	17
		*Genetta abyssinica*	Common genet	LC	8	4	12
	Herpestidae	*Ichneumia albicauda*	White-tailed mongoose	LC	16	10	26
	Felidae	*Felis lybica*	African wildcat	LC	4	6	10
		*Felis serval*	Serval cat	LC	4	3	7
		*Panthera leo*	Lion	VU	2	4	6
Lagomorpha	Leporidae	*Lepus habessinicus*	Abyssinian hare	LC	8	14	22

*LR/lc* Lower risk/least concern, *LC* Least concern, *VU* Vulnerable, *EN* Endangered

Most of the Crested Porcupine (*Hystrix cristata*), Colobus monkey (*Colobus guereza*), Anubis baboon (*Papio anubis*), Abyssinian hare (*Lepushabe essinicus*), Vervet monkey (*Cercopithecus aethiopus*), Common genet (*Genetta abyssinica*), African wild cat (*Felis lybica*), common jackal (*Canis aureus*), Giant root-rat (*Tachyoryctes macrocephalus*), White-tailed mongoose (*Ichneumina lbicauda*) and African civet (*Civettictis civetta*) were medium-sized mammals and spotted hyena (*Crocuta crocuta*), Serval cat (*Felis serval*), Bush pig (*Potamochoerular vatus*), Bushbuck (*Tragelaphus scriptus*), Lion (*Panthera Leo*), and common duiker (*Sylvicapra grimmia*) were the large mammals of the study area.

According to the species composition in the three habitat types, Anubis baboon (*Papio anubis*), Crested Porcupine (*Hystrix cristatea*), and Spotted hyena (*Crocuta crocuta*) were the most common mammals recorded in all three habitat types, while lions (*Panthera leo*) were only recorded in one (grassland habitat). At the family level, Cercopithecidae, Felidae, Bovidae, and Viverridae were the dominant families, while Suidae, Spaclacidae, Hystricidae, Canidae, Hyaenidae, Herpestidae*,* and Leporidae were the least represented families in the study area.

Among the five orders identified;, order Rodentia and order Artiodctayla were represented by two families, the other orders, Primates and Lagomorpha, by one species, and the order Carnivora, by five families. Based on the species, the order Carnivora was represented by the highest number of species (*N* = 8), followed by the Primates and Artiodactayla (*N* = 3 each). The rest of the orders Rodentia and Lagomorpha were represented by two and one species, respectively (Table 2).

The researchers classified the recorded mammals as directly observed and indirect pieces of evidence, and among them, nine of the recorded mammal species, such as Anubis baboon (*Papio anubis*), Vervet monkey (*Cercopithecus aethiopus*), Colobus monkey (*Colobus guereza*), Common jackal (*Canis aureus*), Spotted hyena (*Crocuta crocuta*), Common duiker (*Sylvicapra girmma*), White-tailed mongoose (*Ichneumia albicauda*), Abyssinian hare (*Lepus habessinicus),* and Bushbuck (*Tragelaphus sylvaticus*) were directly observed inside the forest. Three indirectly recorded mammal species, such as Bushpig (*Potamochoerus larvatus*), Giant Root-rat (*Tachyoryctes macrocephalus*), and Common Genet (*Genetta abyssinica*), were identified through patterns of tracks they left behind. Besides these, the Crested Porcupine (*Hystrix cristatea*) and the African civet (*Civettictis civetta*) were identified by the evidence of scats*. *Serval cats (*Felis serval)* and African wildcats (*Felis lybica)* were recorded by the identification of dung. However, the lion (*Panthera leo*) species was assured of its presence by the local villager’s informed witness.

The number of individual observations recorded and the relative frequency of each mammalian species are presented in Table 3 below. During the dry season, the Spotted hyena (*Crocuta crocuta*) has the highest relative frequency of 16.45% (*N* = 51), and the least relative frequency of 0.65% (*N* = 2) was for the Lion (*Panthera leo*). During the wet season, the Anubis baboon (*Papio anubis*) had the highest frequency of 15.73% (*N* = 53), and the Serval cat (*Felis serval*) had the lowest frequency of 0.89% (*N* = 3).

**Table 3:** The relative abundance of medium and large-sized mammalian species in Michole Forest

Common Name	Dry Season		Wet Season	
	Number of Mammals	Relative abundance	Number of mammals	Relative abundance
Anubis baboon	45	14.51	53	15.73
Vervet monkey	31	10.00	36	10.68
Colobus monkey	8	2.58	13	3.86
Common duiker	26	8.39	31	9.20
Bushbuck	5	1.61	11	3.26
Bushpig	11	3.55	17	5.04
Giant root-rat	30	9.68	26	7.72
Crested Porcupine	37	11.94	44	13.05
Common jackal	17	5.48	22	6.53
Spotted hyena	51	16.45	33	9.79
African civet	7	2.26	10	2.97
Common genet	8	2.58	4	1.19
White-tailed mongoose	16	5.16	10	2.97
African wildcat	4	1.29	6	1.78
Serval cat	4	1.29	3	0.89
Lion	2	0.65	4	1.19
Abyssinian hare	8	2.58	14	4.15
Total	310	100	337	100

On the other hand, the study also revealed that the relative abundance of the different species varied between 0–18.84% in the dry season and 0–21.54% in the wet season in the riverine forest habitat, while in the woodland habitat the relative abundance was between 0–15.89% in the dry season and between 0 and 17.92% during the wet season. The relative abundance of the species in the grassland habitat varied from 0 to 26.32% during the dry season and from 0 to 17.1% during the wet season (Tables 4 and 5).

**Table 4:** Relative abundance of species in the three habitat types during the dry and wet seasons

Common name	Relative abundance of species in the three habitat types					
	Riverine Forest		Woodland		Grassland	
	Dry	Wet	Dry	Wet	Dry	Wet
Anubis baboon	16.67	21.54	12.15	17.92	13.85	5.94
Vervet monkey	13.77	15.38	11.21	15.09	0.00	0.00
Colobus monkey	2.17	10.00	4.67	0.00	0.00	0.00
Common duiker	6.52	2.31	9.35	15.09	10.77	11.88
Bushbuck	3.62	0.00	0.00	4.72	0.00	5.94
Bushpig	7.25	7.69	0.00	0.94	1.54	5.94
Giant root-rat	2.90	1.54	9.35	9.43	24.62	13.86
Crested porcupine	12.32	18.46	13.08	0.00	9.23	19.80
Common jackal	5.80	7.69	8.41	11.32	0.00	0.00
Spotted hyena	18.84	10.77	15.89	5.66	12.31	12.87
African civet	0.00	0.00	1.87	9.43	7.69	0.00
Common genet	3.62	0.00	2.80	0.00	0.00	3.96
White-tailed mongoose	0.00	0.00	9.35	0.00	9.23	9.90
African wildcat	2.90	0.77	0.00	1.89	0.00	2.97
Serval Cat	1.45	0.00	1.87	0.00	0.00	2.97
Lion	0.00	0.00	0.00	0.00	3.08	3.96
Abyssinian hare	2.17	3.85	0.00	8.49	7.69	0.00

**Table 5:** Individual observations counted and seasonal variation of medium and large-sized mammalian species among the three habitats

Common name	Species abundance in three habitat types					
	Riverine Forest		Woodland		Grassland	
	Dry	Wet	Dry	Wet	Dry	Wet
Anubis baboon	23	28	13	19	9	6
Vervet monkey	19	20	12	16	0	0
Colobus monkey	3	13	5	0	0	0
Common duiker	9	3	10	16	7	12
Bushbuck	5	0	0	5	0	6
Bushpig	10	10	0	1	1	6
Giant root-rat	4	2	10	10	16	14
Crested porcupine	17	24	14	0	6	20
Common jackal	8	10	9	12	0	0
Spotted hyena	26	14	17	6	8	13
African civet	0	0	2	10	5	0
Common genet	5	0	3	0	0	4
White-tailed mongoose	0	0	10	0	6	10
African wildcat	4	1	0	2	0	3
Serval cat	2	0	2	0	0	3
Lion	0	0	0	0	2	4
Abyssinian hare	3	5	0	9	5	0
Total	138	130	107	106	65	101

In this study, the highest species richness was recorded in the riverine forest habitat (*N* = 25), and the least was recorded in the grassland habitat (*N* = 22). The species richness in various habitats was 25, 23, and 22 for the riverine forest, woodland, and grassland, respectively. The total number of observations for mammalian species in the riverine forest was 268, with woodland (*N* = 213), and grassland (*N* = 166).

### Habitat association and seasonal variation of mammalian species

The study revealed that there was no difference (*χ*^*2*^ = 0.52, *df* = 1, *p* > 0.05) in species composition and richness between the different habitats during both the dry and wet seasons in the study area. Habitat selection of the mammalian species varied seasonally in the study area. The riverine forest had the highest number of species (*N* = 14 and *N* = 11), followed by the woodland (*N* = 12 and *N* = 11), and the grassland habitat (*N* = 10 and *N* = 12) during the dry and wet seasons, respectively.

The highest number of species (*N* = 14) was recorded in the riverine forest during the dry season, and the least was from the grassland habitat (*N* = 10) during the dry season. A total of 268 individual mammals were recorded in the riverine forest habitat, of which 138 were recorded during the dry season and 130 them during the wet season. In total, small numbers of individuals (*N* = 166) were recorded from the grassland habitat. Among the 166 individuals, 65 were recorded during the dry season and the rest, 101, during the wet season. The seasonal abundance of mammals was significantly varied for the three habitats (*χ*^*2*^ = 0.52, *df* = 1, *p* > 0.05): riverine forest (*χ*^*2*^ = 0.36, *df* = 1, *p* > 0.05); woodland (*χ*^*2*^ = 0.52, *df* = 1, *p* > 0.05), and grassland with scattered trees (*χ*^*2*^ = 0.027, *df* = 1, *p* > 0.05).

Differences in species evenness, richness, and diversity between seasons in the stratified vegetation types were higher in the forest area. Application of the Shannon-Wiener information theory revealed that the diversity index and evenness of the mammalian species in the different habitat types of the area were: 0.867 and 0.880 for the riverine forest habitat, 0.887 and 0.920 for the woodland habitat, 0.894 and 0.929 for the grassland habitat, respectively during the wet season (Table 6). During the dry season, the diversity index and evenness of the mammalian species were 0.891 and 0.891 for the riverine forest habitat, 0.902 and 0.936 for the woodland habitat, and 0.877 and 0.922 for the grassland habitat, respectively (Table 6).

**Table 6:** The diversity indices of the medium and large mammalian species in the three habitats of the study area during the dry and wet seasons

Habitat	Season	Number of species	Number of individuals	H	H _max_	Evenness	1-D
Riverine Forest	Dry	14	138	2.351	2.639	0.891	0.891
	Wet	11	130	2.109	2.398	0.880	0.867
Woodland	Dry	12	107	2.325	2.485	0.936	0.902
	Wet	11	106	2.206	2.398	0.920	0.887
Grassland	Dry	10	65	2.122	2.303	0.922	0.877
	Wet	12	101	2.308	2.485	0.929	0.894

## Discussions

A total of 17 species of medium and large-sized wild mammals were identified in the present study. Similar results were recorded in different parts of the country by using similar line transect techniques. For instance, Girma et al. [16] recorded 18 species of medium and large mammalian species in Kaka and Hunkolo Fragnents, Meseret and Solomon [33] recorded 23 medium and large mammalian species from Borena-Sayint Park, south of Wollo, Ethiopia, and Kasso et al. [34] and Gebo et al. [35] identified 21 species in the Chilalo-Glama Forest Priority Area and Faragosa-Fura Landscape, Gamo Zone, Ethiopia, respectively. On the contrary, Lemma and Tekalign [25] documented only a total of eight medium and large mammalian species in the Humbo Community-Based Forest Area, Southern Ethiopia, which is smaller than that of the present study. The order Carnivora was represented by the highest number of families and species during the study period, followed by the orders Artiodactyla and Rodentia. This finding is consistent with the various studies conducted in different places of the country;, where they identified more families of Carnivora [8, 24, 35],

Meseret and Solomon [33] also showed a positive correlation between habitat heterogeneity and animal species diversity. Among the three habitats in the study area, the heterogeneous plant species assemblage available in the woodland and riverine forest contributed to the highest diversity of mammals. The ecological preference and evolutionary adaptation of mammalian species play a role in their occurrence and abundance in different habitat types [36, 37].

Medium and large-sized mammals prefer certain habitat types and, consequently, are not uniformly distributed while foraging. These preferences and the availability of optimal habitat will affect lifetime reproductive success. For African mammals, day-to-day movement between habitats is determined by a diverse set of factors including forage composition, availability, quality, water availability, topography, and soil types [38, 39]. The high abundance of mammalian species in the riverine forest might be due to these factors. The distributions and abundance of medium- and large-sized mammal species were not uniform in the current study area. Riverine forest supported the highest diversity of medium and large-sized mammal species, followed by woodland forest. Open grassland has supported the lowest diversity. Moreover, woodland and riverine forest habitats held a more stable community than open grassland habitats. In this study, woodland and riverine forest habitats have more or less similar distribution and abundance of mammals due to the similarity of vegetation cover, food, and water availability.

The nine medium-sized mammals, Anubis baboon, Crested Porcupine, Vervet monkey, Common Jackal, Common duiker, White-tailed mongoose, African civet, Spotted hyena, Bushpig, and Abyssinian hare, were the most abundant species in the present study area in both wet and dry seasons. But Serval cats and lions were the least abundant species in the present study area. This might be related to the diet and habitat requirements of the animals as identified by Gonfa et al. [40].

This is perhaps due to the high reproductive success, diversified foraging behavior, and high tolerance level of primates to human disturbances. The Anubis baboon was the most abundant mammal in the area. The high species richness of the primates may be associated with the wide distributional range of the species and their more adaptive nature to different habitats. In many areas, this monkey frequents human settlements and feeds extensively on cultivated plants. Several studies have also reported a similar abundance of primates from different parts of Ethiopia [40, 41].

The species is known to be widely distributed in Africa in a wide variety of habitats, from savannah grassland to Afromontane forest. Johnson et al. [42] mentioned that baboons consume a huge variety of items, including roots, tubers, corms, fruits, leaves, flowers, buds, seeds, bark, exudates, cacti, and grasses. *P. anubis* and *C. guereza* have been known to prefer habitats in an altitudinal range between 1200 and 2028 m a.s.l., which is the altitude range of the study area. UNESCO [43] made a similar observation, stating that the distribution of *P. anubis* and *C. guereza* ranged between 1800 and 2600 m a.s.l. This high abundance of species in the study area might be correlated with vegetation cover, altitude, and availability of food.

Spotted hyenas were the most abundant carnivore species recorded in the riverine and woodland habitats of the study area. The Serval cat and lion were the least abundant species of Felidae in the present study area. The average number of individuals recorded per habitat was relatively the same in the three habitats,; however, it was significantly less in woodland and grassland habitats in the dry season. Habitat use and dietary attributes such as composition and quality have a significant effect on animal distribution. As reported by Brnesh *et al.* [44] (2015), the habitat might have limited food and cover to be utilized by the animals.

## Conclusion

The current study's findings shed light on some aspects of the diversity, distribution, and habitat association of medium- and large-sized mammalian species in the Michole forest. It gives baseline information for further studies on mammalian species in the area. Seventeen mammalian species were identified in the study area. Carnivora and primates have the highest abundance, while the order Lagomorpha has the lowest. Spotted hyenas, Anubis baboons, Crested porcupines, and Vervet monkeys were the most abundant species in the study area, while Serval cats and lions were the least abundant. Mammal species distribution and abundance in forests vary due to vegetation types and altitudinal differences. Riverine forest had the highest number of species, followed by woodland and grassland. The distribution and utilization of different vegetation communities by mammal species could be explained in terms of seasonal changes. The habitat preference of the medium- and large-sized mammalian species were influenced by seasonal variations in the quality and abundance of forage. It is possible to conclude that food, water, and protection were decisive in determining the distribution of the mammal species in the present study area. Based on the results of the present study, continuous long-term studies of the ecological aspects of medium and large-sized mammalian species are needed for future conservation measures, and regular assessment and monitoring of the wildlife species are essential in the Michole Forest.

## Data Availability

The data generated and analyzed during the current study are included in the body of this paper.
